# Diet and physical activity interventions in Black and Latina women with breast cancer: A scoping review

**DOI:** 10.3389/fonc.2023.1079293

**Published:** 2023-03-13

**Authors:** Margaret S. Pichardo, Tara Sanft, Leah M. Ferrucci, Yaideliz M. Romero-Ramos, Brenda Cartmel, Maura Harrigan, Ana I. Velazquez, Oluwadamilola M. Fayanju, Eric P. Winer, Melinda L. Irwin

**Affiliations:** ^1^Department of Chronic Disease Epidemiology, Yale School of Public Health, New Haven, CT, United States; ^2^Department of Surgery, Hospital of the University of Pennsylvania, Philadelphia, PA, United States; ^3^Yale Cancer Center, New Haven, CT, United States; ^4^Deparment of Medical Oncology, Yale School of Medicine, New Haven, CT, United States; ^5^Department of Biology, University of Puerto Rico-Humacao, Humacao, PR, United States; ^6^Department of Medicine, Division of Hematology/Oncology, Helen Diller Family Comprehensive Cancer Center, University of California, San Francisco, San Francisco, CA, United States; ^7^Perelman School of Medicine, The University of Pennsylvania, Philadelphia, PA, United States

**Keywords:** breast cancer, Hispanic/Latina women, Black/African American women, energy balance, diet intervention, physical activity intervention, randomized controlled (clinical) trial, survivorship

## Abstract

**Background:**

A growing number of lifestyle interventions are being developed to promote weight loss and adoption of a healthful lifestyles among breast cancer survivors; yet Black and Latina women remain underrepresented.

**Purpose:**

We performed a scoping review of the available peer-reviewed literature to describe and compare the content, design, methods, and primary outcomes of current diet and/or physical activity (PA) interventions after a breast cancer diagnosis among Black and Latina women.

**Methods:**

We queried PubMed, EMBASE, CINAHL, MEDLINE, and Clinicaltrials.gov up to October 1, 2022, to identify all randomized controlled trials of diet and/or PA after diagnosis of breast cancer with a majority (>50%) of Black or Latina participants.

**Results:**

Twenty-two randomized controlled trials were included in this review (five efficacy, twelve pilot, five on-going). Nine trials were among Latinas (two diet, four PA, and three diet/PA), six among Blacks (one PA and five diet/PA) and seven included both populations (five PA and two diet/PA), all of which examined different endpoints. Two of the five efficacy studies achieved their *a priori* outcome (one diet trial improved short term dietary intake; one PA trial achieved clinically significant improvements in metabolic syndrome score), both in Latinas. Eight pilot trials intervened on both diet and PA and three of them found favorable behavioral changes. Three (two for Latinas and one for Blacks) out of the nine diet and PA trials and three (all for Latinas) efficacy trials incorporated a culturally focused approach (i.e., traditional foods, music, Spanish content, bicultural health coaches, spirituality). Overall, four trials, including one efficacy trial, had one-year follow-up data, with three finding sustained behavior change. Electronic/mobile components were incorporated in five trials and one involved informal care givers. Most of the trials were geographically limited to the Northeast USA (n=8, NY, NC, DC, NJ) and Texas (n=4).

**Conclusions:**

Most of the trials we identified were pilot or feasibility studies and of short duration, demonstrating the need for large randomized controlled efficacy lifestyle interventions among Black and Latina breast cancer survivors. Culturally tailored programing was limited but is an important component to incorporate in future trials in these populations.

## Introduction

Breast cancer is the most common type of cancer among women in the United States (US) (1). Historically, Black/African American (herein referred to as Black) and Hispanic/Latina (herein referred to as Latina) women have had lower incidence of breast cancer than Non-Hispanic White (herein referred to as White) women, but this gap is closing (2, 3). Of note, Black women are more likely to be diagnosed with breast cancer at an earlier age (3) and experience a 39% higher disease-specific mortality than White women (4, 5). While Latina women experience lower risk of breast cancer-specific mortality than White women, breast cancer remains the leading cause of cancer death among Latinas (2). Latina women are more likely to be diagnosed with regional or distant breast cancer and tumors with worse prognosis (i.e., Stage IV, larger and hormone receptor negative tumors) compared to White women (6, 7). Further differences exist at the intersection of race and ethnicity, for example among Latinas, Hispanic Black women have higher rates of triple negative breast cancer than Hispanic White women (6). Intervention strategies to improve outcomes in these populations are needed.

Obesity disproportionately burdens Black and Latina women compared to White women (8) and is strongly associated with breast cancer risk (9, 10) and prognosis (11–13). The age-adjusted obesity prevalence from 2013-2014 for Black and Latina women was 53% and 47% compared to 38% for White women (8). Severe obesity is also of concern in Black women; 17% of Black have a body mass index (BMI) over 35 kg/m^2^, compared to 9% and 10% of Latina and White women, respectively (8). Central adiposity is an important risk factor for postmenopausal breast cancer (14, 15) and is associated with hormone receptor positive tumors in Black women (16). Gaining weight before menopause is associated with increased breast cancer incidence (15, 17–19) and risk of recurrence (20, 21), as well as disease-specific and all-cause mortality (21–24). Weight gain after a diagnosis of breast cancer and initiation of adjuvant chemotherapy (25–28) increases risk of recurrence and breast cancer mortality (29). Given this evidence, it is crucial to promote physical activity, a healthy diet, and the avoidance of obesity and weight gain after a breast cancer diagnosis through the adoption of healthy lifestyle behaviors. Current guidelines from the American Cancer Society (ACS) recommend that cancer survivors follow a healthy diet (e.g. low in fat, rich in vegetables, fruits, and whole grains) and attain 150-300 minutes of aerobic exercise and do at least two strength training sessions weekly (30). The most recent American Society of Clinical Oncology (ASCO) guidelines recommend engaging in these behaviors as early as possible after diagnosis (31).

Various studies have examined adherence to the lifestyle recommendations among Black women with breast cancer with results showing low adherence to these (32–34). Nonetheless, to our knowledge, there are no studies of adherence to combined diet and physical activity guidelines among survivors of color. Data derived from studies of predominantly White women with breast cancer suggest that engaging in post-diagnosis, healthy lifestyles, consisting of a high-quality diet and any physical activity, is associated with a reduction in risk of both breast-cancer specific and all-cause mortality (35, 36). Lifestyle interventions consisting of both diet and physical activity counseling may help breast cancer survivors adopt and adhere to the recommended guidelines by providing evidence-based tools for survivors to adopt and maintain healthy behaviors (37). For instance, the Lifestyle, Exercise And Nutrition (LEAN) trial, enrolled 100 breast cancer survivors of whom 91% were White and 9% non-White, demonstrated improvements in body weight *via* an intervention on physical activity and consumption of healthy foods in survivors with breast cancer with an in-person or telephone counseling intervention compared to usual care (38).

In addition to promoting weight loss, physical activity may protect against or ameliorate certain complications from breast cancer treatment. Exercise trials during and after breast cancer treatment has improved lymphedema risk, cancer-related fatigue (39–41), quality of life (42, 43), emotional functioning (39), self-esteem (44), depressive symptoms (45), pain symptoms (39, 46), cardiovascular function (43), muscular strength (43, 44), sarcopenia (41) and age-associated muscle loss (i.e., dynapenia) (41), and chemotherapy completion rates (44). Observational studies in cohorts of predominantly White women have documented a link between diet quality and mortality in cancer survivors (35, 47, 48). For example, a study of 2,317 women (5.6% Black and 2% Latina) with invasive breast cancer participating in the Women’s Health Initiative found that women with a higher quality diet had a 26% lower risk of all-cause mortality and 42% lower risk of death from causes other than breast cancer, although no association was found with breast cancer-specific death (47). In a secondary analysis of the Multiethnic Cohort among 17,330 White, 9,014 Black, 17,595 Latina, 4,992 Native Hawaiian, and 21,239 Japanese American women, higher diet quality—measured by various dietary indices—was associated with lower risk of death from all causes, cardiovascular disease and cancer (48). Among 2,437 women enrolled in the Women’s Intervention Nutrition Study (WINS), where 5.2% of women identified as Black (n=127), 4% as Latina (n=98), and 6% as Asian/Pacific Islander (n==144), there was a 24% higher 5-year relapse-free survival in women who reduced their dietary fat intake compared to the control group (HR: 0.76; 95% CI, 0.6-0.98) (49, 50). However, the Women’s Healthy Eating and Living (WHEL) study conducted among 3,088 women (3.8% Black (n=118), 5.3% Latina (n=165), 3.1% Asian (n=96)), found that a diet high in vegetables, fruits, and fiber and low in fat was not associated with a reduction in additional breast cancer events or mortality (51), demonstrating that uncertainties about the effects of diet on breast cancer outcomes remain.

At present, our knowledge of the benefits of dietary and physical activity interventions for survivors with breast cancer in relation to health outcomes and health-related quality of life is derived from studies targeting mostly White women, with close to 200 lifestyle randomized lifestyle interventions published in this population to date (52). In a systematic review of 17 reviews, Lake and colleagues found that interventions that provided lifestyle counseling and support for both physical activity and diet components, where of longer duration, and were group-based, were the most effective to achieve weight loss and improvements in mental health outcomes among predominantly White breast cancer survivors (52). To better understand the state of lifestyle intervention research in Black and Latina women, we conducted a scoping review to summarize the current state of the evidence (53, 54) of diet and/or physical activity interventions for Black and Latina women after a diagnosis of breast cancer.

## Materials and methods

### Search strategy

**Figure 1:**
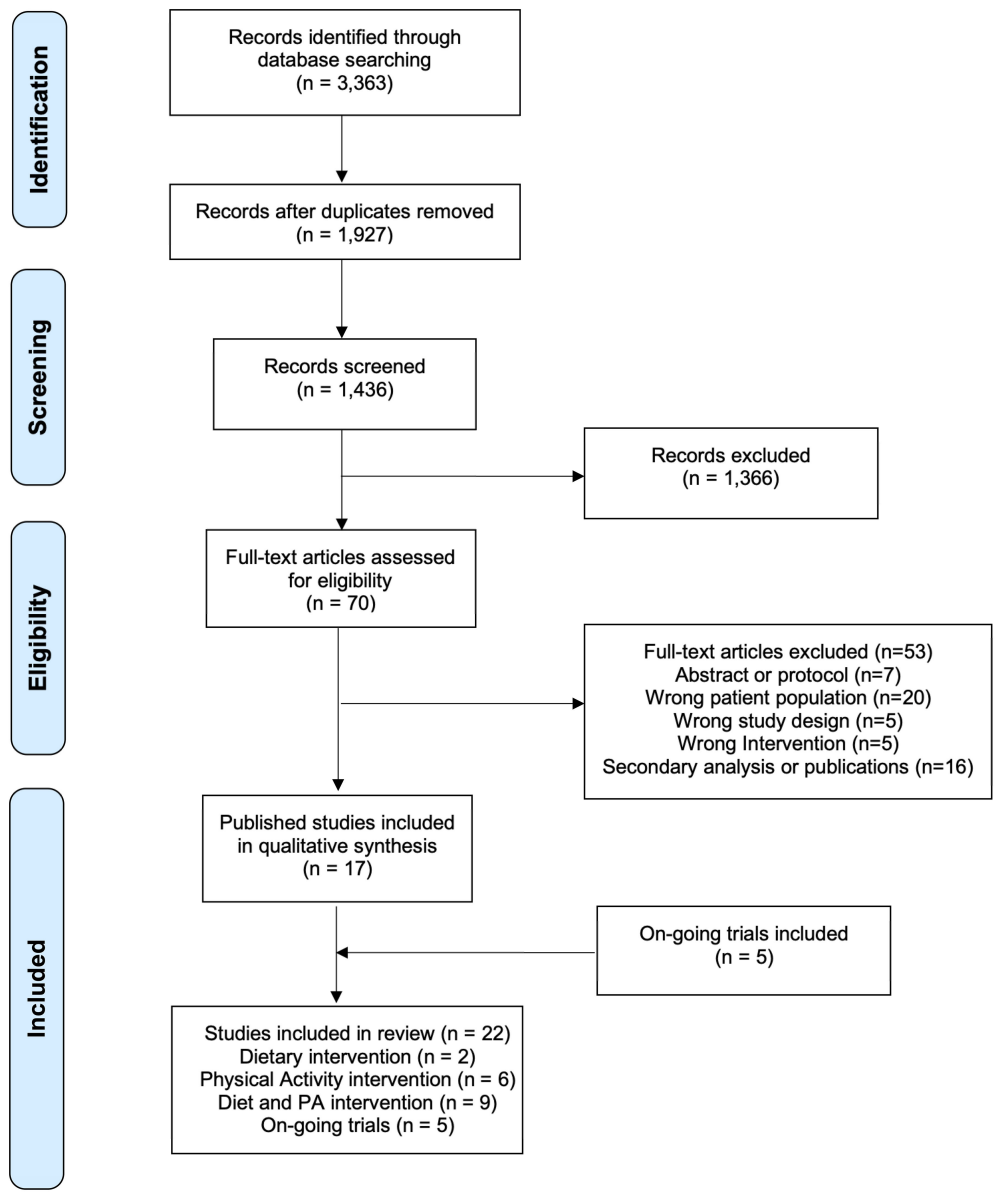
Preferred reporting items for systematic reviews and meta-analysis extension for scoping reviews diagram flow of literature search.

Our scoping review adhered to the guidelines described in the Preferred Reporting Items for Systematic Reviews and Meta-analyses extension for Scoping Reviews (PRISMA-SCR) (55). A structured literature search was conducted through October 2, 2022, without date restrictions, in five databases: PubMed, EMBASE, MEDLINE, CINAHL, and clinicaltrials.gov. A PRISMA diagram summarizing our search and screening results is shown in **Figure 1**. The strategy (**Appendix**) used for our search was adapted from Spark et al. (56) and modified to include four overarching concepts: 1) breast cancer, 2) diet or physical activity intervention, 3) Black or Latina women, and 4) randomized study design. To identify ongoing studies in the clinicaltrials.gov registry, we restricted our search to “breast cancer” disease, studies with a clinical status of “not yet recruiting”, “recruiting’, or “enrolling by invitation”, study types categorized as “interventional (clinical trial)” and used a combination of the following: “diet”, “nutrition”, “physical activity”, “exercise”, “African American”, “Black”, “Hispanic”, “Latina”.

### Trial inclusion criteria

Literature screening was conducted using Covidence Systematic Review Software (Veritas Health Innovation, Melbourne, Australia. Available at www.covidence.org). Our search yielded 3,363 publications (Pubmed, n = 821; MEDLINE (OVID), n = 968; EMBASE (OVID), n = 1,415, CINHAL, n = 159). A total of 1,927 duplicates were excluded leaving us with 1,436 publications (**Figure 1**). For inclusion in this review, the publication had to report on results of a diet and/or physical activity intervention where ≥ 50% of participants identified as a Black or Latina women and had a history of breast cancer. During title and abstract screening, all 1,436 publications were screened by two authors (MSP, YMRR) and 70 studies were identified for full-text review. During full-text review, all studies were reviewed by two authors (MSP, YMRR) and 53 studies were excluded. Any discrepancies between the two reviewers were resolved by discussion among three authors (MSP, YMRR, MI) and by referencing the full text of the manuscript. Exclusion reasons were as follows: publication was a protocol or an abstract (n = 7), study did not include appropriate study population (not breast cancer patient/survivor or participants were not > 50% Black or Latina women; n = 20), study was not a behavioral diet and/or physical activity intervention (n = 5), study was not a randomized controlled trial (n = 5), and study was a secondary analysis or publication from a trial previously included (n = 16). The clinicaltrials.gov registry search resulted in five trials registered as on-going that did not have publications on primary outcomes.

## Results

We identified a total of twenty-two diet and/or physical activity intervention articles that met our inclusion criteria. A brief description of key characteristics of published studies is shown in **Table 1**. A detailed description of completed studies is provided in **Table 2**. A summary of completed trials with published findings is presented in **Table 3**. A brief description of trial characteristics and primary outcomes for registered ongoing and withdrawn trials is provided in **Table 4**.

**Table 1:** A brief description of key characteristics of published studies (N = 17).

Author	Intervention content	Delivery method	No. of Participant/Race or Ethnicity	Disease stage	Study design	Linguistic and/or culturally tailored?	Long-term follow up (time frame)?	Primary outcome
Greenlee, 2015 (57)	Diet	In-person	70/Latina	0-III	Efficacy	Yes: Content & language	Yes (6 and 12 months)	Intake of fruits and vegetables (F&V) servings; Percent calories from fat
Zuniga, 2018 (58)	Diet	In-person	64 Latina, 53 White, 8 Other	0-III	Efficacy	No	No	Adherence to anti-inflammatory diet
Dieli-Conwright, 2018 (59)	Physical Activity	In-person	55/Latina, 4/Black, 26/White, 15/Asian/Pacific Islander	0-III	Efficacy	No	No	Metabolic syndrome z-score
Moadel, 2007 (60)	Physical Activity	In-person	54/Black, 40/Latina, 29/White, 5/Other	I-IV	Efficacy	No	No	Quality of life score
Mama, 2018 (14)	Physical Activity	In-person; At home	89/Latina	I-IV	Pilot	Yes: Content & language	No	Social cognitive theory measures; Minutes of physical activity
Taylor, 2018 (61)	Physical Activity	In-person	33/Black	Not reported	Pilot	No	No	Psychological and functional outcomes
Lee, 2020 (17)	Physical Activity	In-person	22/Latina, 2/Black, 4/White, 2/Asian/Pacific Islander	I-III	Pilot	No	No	Feasibility
Soltero, 2022 (62)	Physical Activity	In-person	13/Latina, 1/Black, 5/White, 1/Other	0-III	Pilot	No	No	Overall daily steps, BMI, body fat
Stolley, 2017 (63)	Diet and Physical Activity	In-person	246/Black	I-III	Efficacy	Yes: Content only	Yes (12 months)	5% Weight loss
Ferrante, 2018 (64)	Diet and Physical Activity	Electronic (Spark People website)	37/Black	0-III	Pilot	No	Yes (12 months)	5% Weight loss
Valle, 2017 (65)	Diet and Physical Activity	Electronic (Email, Mobile application, Website)	45/Black	I-III	Pilot	No	Yes (6 months)	Weight gain prevention
Greenlee, 2013 (66)	Diet and Physical Activity	In-person	33/Latina, 9/Black,	0-III	Pilot	Yes: Language only	Yes (12 months)	5% Weight loss
Paxton, 2017 (67)	Diet and Physical Activity	Electronic (Email; Individualized website)	59/Black, 8/Latina, 4/Other	0-IV	Pilot	No	No	Meeting exercise/dietary American Cancer Society recommendations
Sheppard, 2016 (68)	Diet and Physical Activity	In-person	31/Black	0-III	Pilot	Yes: Content only	No	5% Weight loss
Buscemi, 2020 (69)	Diet and Physical Activity	Electronic (Mobile application)	80/Latina	0-III	Pilot	Yes: Content & language	No	Dietary intake; Minutes of physical activity
Crane, 2021 (31)	Diet and Physical Activity	Electronic (Telephone)	45/Latina	Not Reported	Pilot	Yes: Content & language	No	Dietary intake; Minutes of physical activity; Feasibility; Acceptability
Allicock, 2021 (32)	Diet and Physical Activity	Electronic (Mobile application)	22/Black	Not Reported	Pilot	No	No	Feasibility

**Table 2:** Description of completed randomized controlled trials focused on dietary and/or physical activity behavioral changes in Black and Latina breast cancer survivors; sample, intervention, and methodology characteristics (N = 17).

Trial name, First Author, Location (Type of Behavior)	Study Design	Sample Characteristics	Intervention \ Characteristics	Study Measures	Primary Outcomes
Diet Interventions (n= 2 efficacy trials)					
*¡Cocinar para su Salud!*, Greenlee, 2015 (57), New York (Diet) *Other publications* (70–72)	RCT, 2-arm, efficacy	Group (N): 70 I (34); C (36) Sample: Survivors; -77% Dominican -7% Puerto Rican -7% Ecuadorian Stages: 0 - III Mean time since diagnosis: 3.4 y Mean age: 56.6 y Mean BMI: 30.9 kg/m^2^ Recruitment: oncology clinics at academic cancer center	Theory: Stages of Change Construct and Social Cognitive Theory Duration: 3mo. Delivery: All: Nutrition educational printed material in Spanish I: Nine in-person Saturday classes lasting 1.5 to 3.5 hours: - 4 nutrition education with a registered dietitian - 3 cooking classes with Latina chef - 2 food shopping field trips to local supermarket and greenmarket C: Usual care Contact: Monthly calls with a dietitian during 3mo. Follow-up: 6mo., 12mo.	Physical Activity measure: Block Physical Activity Screener Dietary measure: Three 24-hour recall assessments (2 weekdays, 1 weekend day, one in person at baseline, two over the phone).	Change in intake of servings of fruits and vegetables % Calories from fat
Zuniga, 2018 (58), Texas (Diet) *Other publications* (73)	RCT, 2-arm, efficacy	Group (N): 153 I (76); C (77) Sample: Survivors; -51.2% Latina -42.4% Non-Hispanic White -6.4% Other Stages: 0 - III Mean time since diagnosis: 2 y Mean age: 57 y Mean BMI: Not Reported Recruitment: Not Reported	Theory: Not Reported Duration: 6mo. Delivery: I: Monthly in-person group nutrition workshops about anti-inflammatory foods and cancer recurrence. - Didactic portion and cooking demonstrations with chef trained in AI food preparation, a tasting, and interactive discussion with participants and research staff. - Encouraged to attend 6 monthly workshops - Received paper copies of presentation material - Received motivational interviewing C: Usual care - Monthly American Institute for Cancer Research informational brochures Contact: Monthly calls with trained patient navigators for 6 mo. Follow-up: none	Physical activity measure: none Dietary measure: 14-item Mediterranean diet assessment tool and a 3-day food record (two weekdays and one weekend day) prior to assessment	Change in adherence to anti-inflammatory dietary pattern
Physical Activity Interventions (n= 2 efficacy trials; n= 4 pilot/feasibility trials)					
Dieli-Conwright 2018 (59), California (Physical Activity) *Other publications* (74)	RCT, 2-arm, efficacy	Group (N): 100 I (50); C (50) Sample: Survivors; -55% White Latina -26% Non-Hispanic White -4% Black -15% Asian/Pacific Islander Stages: 0 – III Mean time since diagnosis: 6.2 mo. Mean age: 53.5 y Mean BMI: 33.5 kg/m^2^ Recruitment: academic cancer center and affiliate public hospital	Theory: American Cancer Society (ACS) exercise guidelines for cancer survivors. Duration: 4mo. Delivery: All: Asked to maintain dietary behaviors during the study period I: Supervised, one-on-one training provided by a certified cancer exercise trainer. - 3 weekly sessions of resistance and aerobic exercise lasting ~80 mins for sessions 1 and 3 and of aerobic exercise of ~50 mins long for session 2. - Wore Polar heart monitors during sessions. C: Wait-listed - Wore a daily accelerometer Contact: Weekly for 6mo. Follow-up: 7mo.	Physical activity measure: Physical activity history measured at baseline with interviewer-administered validated questionnaire. Determined maximal oxygen update with a single stage submaximal treadmill test. Assessed maximal voluntary strength (one-repetition maximum) for chest press, latissimus pulldown, knee extension and knee flexion using the 10-repition maximum method. Dietary measure: 2 week days and 1 weekend day dietary records at baseline, post-intervention and at 3 m follow up for I group only.	Metabolic syndrome z-score based on the following variables: -Waist circumference -Systolic and diastolic blood pressure -HDL cholesterol -Triglycerides -Glucose
Moadel 2007 (13), New York (Physical Activity)	RCT, 2-arm, efficacy	Group (N):128 I (108); C (44) Sample: Patients and survivors; -42% Black -31% Latina -23% White -4% Other Stages: I - IV Mean time since diagnosis: 1.1 y Mean age: 54.8 y Mean BMI: Not Reported Recruitment: Oncology clinics at academic medical center and private clinics	Theory: Not Reported Duration: 3mo. Delivery: I: immediate intervention 12 in person, 1.5-hour Hatha yoga sessions with certified instructor. Participants able to attend >1 class/wk. C: Wait-listed Contact: Baseline and after 3mo. Follow-up: none	Quality of Life measure: The Functional Assessment of Cancer Therapy (FACT)	Change in Quality of Life
Project *VIVA*! Mama 2018 (14), Texas and Puerto Rico (Physical Activity) *Other publications* (75)	RCT, 3-arm, pilot	Group (N):89 I (59); C (30) Sample: survivors -45 Mexican American -44 Puerto Rican Stages: I-IV Mean time since diagnosis: Not Reported Mean age: 58.5y Mean BMI: 31.0 kg/m^2^ Recruitment: Oncology clinics at academic medical center.	Theory: Social cognitive theory Duration: 4mo. Delivery: All: Twice a week home-based exercise program consisting of aerobic exercise, muscular strength, and flexibility training. - Intensity and duration were individually tailored. - Two sets of resistance band, a pedometer. an exercise book and video - Group exercises were held once a month. I-1: Culturally adapted group (n=30) - Culturally relevant images, messages, and examples to Latina breast cancer survivors - Information on self-efficacy, social modeling, and social support I-2: Standard exercise group (n=29) C: Wait-listed Contact: Biweekly phone calls for 4mo. Follow-up: 6mo.	Physical Activity and Sedentary Time: The International Physical Activity Questionnaire (IPAQ) short form to measure Physical Activity and sedentary time over the past seven days. Sedentary behavior: Past-day Adults’ Sedentary Time (PAST) Questionnaire. SCT variables: a range of scales to measure exercise self-efficacy, barriers self-efficacy, social modeling of Physical Activity, and social support for exercise.	Compare culturally adapted vs standard intervention on the following: -Social cognitive theory measures -Physical activity -Sedentary time
Taylor 2018 (16), Washington, DC (Physical Activity)	RCT, 2-arm, pilot	Group (N):33 I (18); C (15) Sample: Survivors -100% Black Stages: Not Reported Mean time since diagnosis: (I) 9.3 y and (C) 6.5 y Mean age: (I) 54.9 y and (C) 52.6 y Mean BMI: (I) 33.8 kg/m^2^ and (C) 33.9 kg/m^2^ Recruitment: Oncology clinics at academic medical center	Theory: Not Reported Duration: 2 mo. Delivery: I: Eight weekly restorative yoga classes of 75 minutes per session led by a certified yoga instructor at Howard University. - Yoga breathing techniques Pranayama. C: Wait-listed Contact: Baseline and after 2mo. Follow-up: none	Fatigue: 9-item self-reported Brief Fatigue Inventory scale, to assess fatigue and impact of fatigue on daily functioning. Insomnia: 7-item Insomnia Severity Index (ISI) measure to evaluate the perceived severability of clinically significant insomnia over 2 wks. Depression: Center for Epidemiologic Studies Short Depression Scale (CES-D-R 10). 10-items self-reported, to measure depressive symptomatology. Perceived stress: 4-item Perceived Stress Scale (PSS). Yoga Satisfaction: Assess participants opinion of the yoga program.	Changes on psychological and functional outcomes
Lee, 2021 (17), California (Physical Activity) *Other publications:* (76–79)	RCT, 2-arm, pilot	Group (N): 30 I (15); C (15) Sample: Patients -13% Non-Hispanic White -73% Latina -7% Black -7% Asian/Pacific Islander Stages: I-III Mean time since diagnosis: 8 wks from completing (neo)adjuvant Mean age: 46.9 y Mean BMI: I (33.1 kg/m^2^); C (30.1 kg/m^2^) Recruitment: Oncology clinics at academic medical center and affiliate public hospital	Theory: Not Reported Duration: 8 wks Delivery: All: Maximal cycling protocol that included 10 W increase in workload every 60s, starting at 40 W while maintaining 60 rpm to measure their VO2max and PPO (highest power output generated during a maximal cycling test). I: Eight weekly HITT supervised sessions by a certified exercise trainer on a stationary bike. C: Wait-listed Contact: Baseline and after 8 wks. Follow-up: none	Physical Activity measure: Timed up and go (TUG), the 30-s sit-to-stand (30STS) test, the Margaria-Kalamen stair climb test, and the 6-min walk test (6MWT).	Feasibility of utilizing HIT, measured using the average minutes of weekly activity and the number of sessions attended
Soltero 2022 (22), Arizona (Physical Activity)	RCT, 2-arm, pilot	Group (N): 20 Arm 1(10); Arm 2 (10) Sample: Survivors -65% Latina -25% Non-Hispanic White -5% Black -5% Mixed race/ethnicity Stages: 0-III Mean time since diagnosis: 2 wks to 10 y past primary treatment Mean age: Arm 1 (49.6 y), Arm 2 (53.2 y) Mean BMI: Arm 1 (31.0 kg/m^2^) Arm 2 (31.1 kg/m^2^) Recruitment: Oncology clinic and dissemination through the cancer support community.	Theory: Not Reported Duration: 8 wks Delivery: All: Used a 7-day pedometer and a Tanita TBF-310 body composition analyzer. Twice a week classes. Arm 1: Latin dance classes - Provided by Latin dance instructors, included basic salsa, merengue, chacha and bachata. Arm 2: Qigong/Tai Chi classes - Provided by Tai Chi Easy instructors. There were 7 basic core exercises, 10 additional movements and standardized opening and closing movements. C: none Contact: Baseline and after 8 wks. Follow-up: none	Physical Activity measure: 7-day pedometer protocol	Overall daily steps BMI Percent body fat
Combined Diet/Physical Activity Interventions (n= 1 efficacy trial; n= 8 pilot/feasibility trials)					
Moving Forward, Stolley 2017 (9), Chicago (Diet and Physical Activity) *Other publications* (80–82)	RCT, 2-arm, efficacy	Group (N): 246 I (125); C (121) Sample: Survivors; -100% Black Stages: I – III Mean time since diagnosis: 6.7 y Mean age: 57.5 y Mean BMI: 36.1kg/m^2^ Recruitment: Cancer registry	Theory: Socioecological model Duration: 6mo. Delivery: I: Interventionist-guided program - Class 1: twice-weekly, 90 min. in-person, supervised group exercise sessions followed by 45-60 min learning modules; text messaging counseling - Class 2: standalone, 60 min. exercise session. Provided program binder. C: Self-guided program - Received program binder Contact: Baseline and after 6mo. Follow-up: 12mo.	Physical Activity measure: Modified Activity Questionnaire to determine frequency and duration of moderate and vigorous activity Dietary measure: Block 2005 Food Frequency Questionnaire to determine intake of energy, fruits and vegetables, fat, fiber, meat, and added sugars.	5% weight loss
Ferrante 2018 (23) New Jersey (Diet and Physical Activity, eHealth tools) *Other publications* (83)	RCT, 2-arm, pilot	Group (n): 37 I (20); C (17) Sample: Survivors -100% Black Stages: 0-III Mean time since diagnosis: 6.6 y Mean age: 61.5y Mean BMI: 37.7 kg/m^2^ Recruitment: Oncology clinics at academic medical center	Theory: Not Reported Duration: 6 mo. Delivery: I: Instructed to self-monitor diet weekly using SparkPeople website and physical activity levels daily using Fitbit device. - Active phase: Weekly motivational reminders to log into website for 3mo. - Maintenance phase: Additional 3mo. without reminders. C: Wait-listed Contact: Baseline, at 3mo. and after 6mo. Follow-up: 9mo. and 12mo.	Physical Activity measure: Direct data downloads from the Fitabase research platform provided Physical Activity levels. Dietary measure: Caloric intake was quantified by 24-hour diet recall administered by research assistant using the Sparkpeople.com food diary tool.	5% weight loss
Valle 2017 (25), North Carolina (Diet and Physical Activity, eHealth tools)	RCT, 3-arm, pilot	Group (n): 45 I (34); C (11) Sample: Survivors -100% Black Stages: I-III Mean time since diagnosis: 3.1 y Mean age: 53 y Mean BMI: 33.9 kg/m^2^ Recruitment: Hospital based-registry/cancer survivorship cohort, oncology clinics at academic medical center, local tumor registry, advertising at community-based events and social media.	Theory: Self-regulation theory of eating and exercise behaviors to prevent weight gain and two additional frameworks used in STOP Regain and SNAP which emphasized daily self-weighing. Duration: 6 mo. Delivery: All (intervention): - In-person individualized sessions - Bluetooth and Wifi-enabled wireless scale (Withings WS-30, Cambridge, MA) - Mobile app with graphs and weight trends - Weekly emails with tailored feedback on weight data. I-1: Self-regulation intervention with objective activity monitoring (n=11) Activity tracker (Withings Pulse, Cambridge, MA) I-2: Self-regulation intervention only (n=13) - Encouraged to daily track their activity in addition to weighing themselves C: Wait-listed Follow-up: 6mo.	Physical Activity measure: Paffenbarger Activity Questionnaire (PAQ). Dietary measure: Automated Self-Administered 24-Hour Dietary Recall (ASA-24).	Weight gain prevention
*La Vida Activa/*An Active Life Greenlee 2013, (26), New York (Diet and Physical Activity)	RCT, 2-arm, pilot	Group (n): 42 I (22); C (20) Sample: Survivors; -79% Latina -21% Black Stages: 0 - III Mean time since diagnosis: 1.2 y Mean age: 51 y Mean BMI: 33.2 kg/m^2^ Recruitment: oncology clinics at academic medical center	Theory: Not Reported Duration: 6 mo. Delivery: I: Curves Weight Management Program curriculum available to the public. Program includes a 30-minute exercise circuit and a high vegetable/low-fat/calories-restricted diet. C: Wait-listed Contact: Baseline, 3mo and at 6mo. Follow-up: 9mo and 12mo.	Physical Activity measure: Self-administer adaption of the Kaiser Physical Activity Survey Dietary measure: Spanish version of the Block Questionnaire.	5% weight loss at 6 mo.
ALIVE, Paxton 2017 (27), Texas (Diet and Physical Activity, eHealth tools)	RCT, 2-arm, pilot	Group (n): 71 Arm 1 (34); Arm 2 (37) Sample: Survivors -83% Black -11% Latina -6% Mixed race/ethnicity Stages: 0 - IV Mean time since diagnosis: 8.4 y Mean age: 52.2 y Mean BMI: 30.8 kg/m^2^ Recruitment: North Texas metropolitan area	Theory: Social cognitive theory, goal-setting theory, social marketing, and transtheoretical theory Duration: 3 mo. Delivery: All: Weekly emails and links to an individualized website with behavior change strategies tailored to their specific needs and specific to their track. $20 incentive for completing each assessment. Arm 1: Physical activity track: encouraged to meet exercise recommendations (≥150 min of moderate to vigorous Physical Activity per week) Arm 2: Dietary track - Sub-track 1: F&V: encouraged to meet or exceed recommended F&V consumption (≥3.5 cup svgs of F&V) - Sub-track 2: Fats and added sugar: encouraged to decrease consumption of saturated and trans fats and carbohydrates (≤50g/day of added sugars and ≤10% of calories from saturated fats) C: none Contact: Weekly for 3mo. Follow-up: none	Physical Activity measure: Physical Activity Questionnaire (PAQ) adapted from the Cross-Cultural Activity Participation Study (CAPS) Questionnaire. Dietary measure: 35-item NHANES questionnaire	Meet exercise recommendations Meet dietary recommendations
Stepping STONE Sheppard 2016 (28), Washington, DC (Diet and Physical Activity)	RCT, 2-arm, pilot	Group(n): 31 I (15); C (16) Sample: Survivors; -100% Black Stages: 0-III Mean time since diagnosis: Not Reported Mean age: 54.7 y Mean BMI: I (35.2 kg/m^2^); C (37.4 kg/m^2^) Recruitment: Two local hospitals and community outreach in the Washington, DC metropolitan area.	Theory: Theory of planned behavior and social cognitive theory Duration: 3 mo. Delivery: I: Biweekly 90-min group sessions (30 min supervised group exercise and 60 min education sessions) co-led by a physiologist and a nutritionist. - 6 individual telephone coaching sessions led by a survivor coach. - Received a pedometer, notebook and individualized step goals that gradually increased to 10,000 steps/day for 12 wks. C: Usual care - NCI booklet “Facing Forward Life after Cancer Treatment” Contact: Baseline and after 3mo. Follow-up: none	Physical Activity measure: International Physical Activity Questionnaire Short Form (IPAQ-SF) Dietary measure: Intervention participants were instructed to record daily food/beverage intake.	5% weight loss
MyHealth Smartphone Intervention Buscemi 2020 (29), Chicago (Diet and Physical Activity, eHealth tools) *Other publications* (84)	RCT, 2-arm, pilot	Group(n): 80 Arm 1 (40); Arm 2 (40) Sample: Survivors -100 % Latina Stages: 0-III Mean time since diagnosis: 15.50 m Mean age: 53.54 y Mean BMI: Not Reported Recruitment: Two large academic medical centers in the Chicago metropolitan area and a local community-based organization	Theory: Followed a telecoaching adapted from a model of supportive accountability to promote optimal adherence Duration: 6 wks Delivery: Mobile application on personal phone or borrowed study appointed smartphone All: 15-20 mins telecoaching calls until wk 2 For wks 3-5: - If used app <=90mins, received additional telecoaching calls - If used app >90 mins, received reinforcing text message Arm 1: *My Guide* application (health-related quality of life) Arm 2: *My Health* application for culturally appropriate lifestyle promotion: C: None Contact: Baseline and after 6 wks Follow-up: 8 wks.	Physical Activity measure: 7-item International Physical Activity Questionnaire Dietary measure: 23-item Brief Dietary Assessment Tool for Latinas	Dietary intake Physical activity Breast cancer symptom burden Health-related quality of life domains (breast cancer, physical, emotional, functional well-being)
*Nuestra Salud*/ Our Health, Crane 2020 (31), Arizona (Diet and Physical Activity, eHealth tools)	RCT, 2-arm, pilot	Group(n): 45 dyads I (28); C (17) Sample: Survivors; -100 % Latina Stages: Not Reported Mean time since diagnosis: Not Reported, completed primary treatment Mean age: 64.35 y Mean BMI: I (31.34 kg/m^2^); C (27.08 kg/m^2^) Recruitment: Latina cancer survivors from the southern Arizona community, oncology clinics at academic medical center, and a support group in the Arizona, US-Sonora, Mexico border region.	Theory: Social Cognitive Theory Duration: 12 wks Delivery: All: $25 gift card after study completion. I: A 12 weekly Symptom Management and Lifestyle Intervention (SMLI) telephone-based (20 to 30 mins) coaching sessions with trained bicultural health coach in either English or Spanish using the electronic health and intervention platform (eHIP). - Printed materials from the Symptom Management and Survivorship Handbook developed by the authors (SMSH). - Fitbit as a strategy for self-monitoring. - Specific, Measurable, Attainable, Relevant, and Timely (SMART) goals composed of increasing the number of steps per day (daily activity); servings of F&V or whole grains per day; reduction of calories from added sugars, fat, and processed and red meat; and reduction in alcohol consumption. C: Usual care Contact: Baseline and after 12 wks. Follow-up: none	Physical Activity measure: A Spanish-translated version of the Women’s Health Initiative (WHI) Physical Activity Questionnaire. Dietary measure: A 19-item NCI Dietary Screener Questionnaire 18-item United States Department of Agriculture Food Security Questionnaire	Feasibility & acceptability Efficacy in dietary and Physical Activity adherence Efficacy in symptom improvement
Mobile Health, Allicock 2020 (32), Dallas, Texas (Diet and Physical Activity, eHealth tools)	RCT, 2-arm, pilot	Group (n): 22 I (13); C (9) Sample: Survivors; -100% Black Stages: Not Reported Mean time since diagnosis: Not Reported, ≥6 months since completion of breast cancer treatment Mean age: 52.23 y Mean BMI: I (33.26 kg/m^2^); C (38.25 kg/m^2^) Recruitment: Word of mouth and flyers in Dallas, Texas metropolitan area.	Theory: Social cognitive theory and control theory Duration: 4 wks Delivery: All: ActiGraph wGT3X-BT accelerometer to use for seven consecutive days at baseline, 4 wks, and 8 wks post-baseline. $30 compensation for each of the three study visits and could earn up to an additional $60 for completing 80% or more of the ecological momentary assessments. I: Completed three types of ecological momentary assessments (daily diary, random sampling, event sampling) through the Creating Healthy Actions through Technology (CHAT) app. - Received tailored messages as feedback to their responses. C: Usual care Contact: Baseline and after 4 wks. Follow-up: 8 wks	Physical Activity measure: Behavioral Risk Factor Surveillance System (BRFSS) physical activity questionnaire Dietary measure: 15-item questionnaire, The National Health Interview Survey 2000	Feasibility (i.e., engagement and acceptability) Efficacy of CHAT in behavioral and health outcomes

**Table 3:** Findings of completed randomized controlled trials focused on dietary and/or physical activity behavioral changes in a Black and Hispanic/Latina breast cancer survivors (N = 17).

Trial name, First author, location (Type of behavior)	Findings		
	Feasibility	Change from baseline to post-intervention	Change from baseline to follow-up
Diet Interventions			
Efficacy trials (n=2)			
*¡Cocinar Para Su Salud!*, Greenlee 2015 (1), New York *Other publications* (70–72) (Diet)	End-of-I: -12 wks, I 82/%; C 100% retention Post-I follow-up: 3mo., I 91% and C 100% retention 6mo., I 88/% and C 86% retention 12mo., I 85% and C 80% retention Data analysis: Excluded lost to follow-up	Adjusted means: -All fruit & vegetables, svg: (I) +1.1 vs. (C) -0.3, p=0.05 -Targeted fruit & vegetables, svg: (I) +2.0 vs. (C) +0.2, p=0.004 -Daily total caloric intake (kcal): (I) -672.9 vs. (C) -92.4, p<0.001 -% Fat of daily total energy: (I) -7.1 vs. (C) -1.6, p=0.01	At 6mo. -All fruit & vegetables, svg: (I) +2.0 vs. (C) -0.1, p= 0.005 -Targeted fruit & vegetables, svg: (I) +2.7 vs. (C)+0.5, p=0.002 -Daily total caloric intake (kcal): (I) -562.9 vs. (C) -61.6, p<0.001 -Total fat % of daily total energy: (I) -7.5 vs. (C) -4.4, p= not significant (ns) At 12mo. -All fruit & vegetables, svg: (I) +2.0 vs. (C) -0.4, p= <0.01 -Targeted fruit & vegetables, svg: (I) +2.3 vs. (C) -0.1, p= <0.01 -Daily total caloric intake (kcal): (I) -121.9 vs. (C) 9.3, p= ns -Total fat % of daily total energy: (I) -2.2 vs. (C) -2.1, p= ns
Zuniga, 2018 (5), Texas *Other publications* (73) (Diet)	End-of-I: 6 mo., I 79%; C 84% retention Post-I follow-up: none Data analysis: Excluded lost to follow-up	Marginal means ± standard error (SE) - Mediterranean diet score: (I) +1.6 (0.2) vs. (C) +0.02 (0.2), p<0.001 -Spices and herbs score: (I) +1.9 (0.3) vs. (C) +0.04 (0.2), p<0.001	None
Physical Activity Interventions			
Efficacy trials (n=2)			
Dieli-Conwright 2018 (7)^a^, California *Other publications* (74) (Physical Activity)	End-of-I: 4 mo., I 96%; C 90% retention Post-I follow-up: 3 mo., I 92%; C 90% retention Data analysis: Excluded participants lost to follow-up	- Metabolic syndrome, % of participants: - Baseline: (I) 78% vs. (C) 76%, p=0.27 - Post-intervention: (I) 15% vs. (C) 80%, p<0.004	-Metabolic syndrome, participants in exercise group only: 15%
Moadel 2007 (13), New York (Physical Activity)	End-of-I: 12 wks, 69% retention Post-I follow-up: 3mo., I 78%; C 79% retention 6mo., Not Reported Data analysis: Intention To Treat analysis, subgroup analysis for patients not on chemotherapy	- Overall Quality of Life: δ = -0.09; 95%CI = 8.05, 2.63 - Social well-being: δ = -0.22, 95%CI = 3.78, -0.36 - Physical well-being: δ = 0.07; 95%CI = -1.29, 3.05 - Functional well-being δ = -0.06; 95%CI = -3.29, 1.60 - Emotional well-being: δ = -0.07; 95%CI = -2.33, 0.99	Not Reported
Pilot/feasibility trials (n=4)			
Project VIVA! Mama 2018 (14), Texas and Puerto Rico *Other publications* (75) (Physical Activity)	End-of-I: 16 wks, retention not reported Post-I follow-up: 6mo., Not Reported Data analysis: Assessment of completers only	- Exercise self-efficacy: I vs C, *F* (1,77): 9.17, p=0.003 - Moderate physical activity: I vs. C, *F* (1,76): 7.66, p=0.007 - Vigorous physical activity: I vs. C, *F* (1,76): 6.47, p=0.013 - Total Physical Activity: I vs. C, *F* (1,76): 9.32, p=0.003	Not Reported
Taylor 2018 (16)^b^, Washington, DC (Physical Activity)	End-of-I: 2mo., 60% retention Data analysis: Assessment of completers only	Baseline and Follow-up Mean (standard deviation (SD)) - Sleep quality: (I)10.18 (8.74) and 7.89 (7.17) vs. (C) 7.56 (6.82) and 6.20 (7.11), p = 0.890 - Fatigue: (I) 3.48 (2.34) and 1.85 (1.61) vs. (C) 2.50 (2.71) and 2.10 (2.86), p = 0.750 - Depression: (I) 8.79 (4.23) and 4.78 (3.56) vs. (C) 7.08 (5.38) and 6.91 (5.86), p < 0.01 - Perceived stress: (I) 6.00 (2.48) and 5.22 (2.17) vs. (C) 5.08 (3.06) and 4.45 (3.39), p = 0.770 - Adherence was 61% for the yoga group	None
Lee, 2021(17) California (Physical Activity) *Other publications* (76, 77, 79)	Adherence to 70% of sessions (17/24) End-of-I: 9 wk, I 100%; C 100% retention, 82.3% mean adherence Post-I follow-up: None - Data analysis: Not Reported	- The amount of overall physical activity was not statistically different between the HIIT group (480.9 ± 85.3 Metabolic equivalent (MET)/week) and the control group (441.9 ± 93.2 METs/week).	None
Soltero 2022 (22), Arizona (Physical Activity)	End-of-I: 8 wks, 100 % retention for both groups. Post-I-follow-up: none Data analysis: included post-intervention data collection assessments	- Physical activity level: Increased from T1 to baseline to T2 to post-intervention when examining both arms together but the change was not significant (Cohen’s d= 0.07). - Body composition: No significant changes from T1 to baseline to T2 to post-intervention (Cohen’s d= 0.04 and 0.36, respectively).	Not Reported
Combined Diet & Physical Activity Interventions			
Efficacy trials (n=1)			
Moving Forward, Stolley 2017 (9), Chicago *Other publications* (80–82), (Physical Activity)	End-of-I: 6 mo., I 89%; C 84% retention Post-I follow-up: 12 mo., I 86%; C 83% retention Data analysis: Excluded participants lost to follow-up	- Weight loss, %: (I) 3.6 vs. (C) 1.4, p<0.001 - Moderate physical activity, mins/wk: (I) 98.4 vs. (C) 60.6, p= 0.298 - Vigorous physical activity, mins/wk: (I) 17.4 vs. (C) 2.4, p=0.03 - Daily energy intake, kcal: (I) -563.9 vs. (C) -262.4, p=0.004 -Fiber, g/1,000kcal: (I) 3.24 vs. (C) 0.91, p<0.001 - Added sugars, tsps: (I) -6.98 vs. (C) -3.85, p=0.035	-Weight loss, %: (I) 2.6 vs. (C) 1.6, p=0.05 -Moderate physical activity: (I) 97.8 vs. (C) 77.4 p=0.596 -Vigorous physical activity: (I) 14.4 vs (C) -3.00, p=0.014 -Daily energy intake, kcal: (C) -576.0 vs. (2) -353.9, p=0.037 -Fiber, g/1,000kcal: (I) 1.75 vs (C) 0.78, p=0.046 -Added sugars, tsps: (I) -7.25 vs. (C) 11.4, p=0.030
Pilot/feasibility trials (n=8)			
Ferrante 2018 (23) New Jersey *Other publications* (83), (Diet and Physical Activity, eHealth tools)	End-of-I: 6 mo., 97.1% retention Post-I follow-up: 12 mo., 88.6% retention Data analysis: Intention To Treat, excluded 2 participants from intervention group that did not meet eligibility criteria after randomization	Baseline and Follow-up (6 mo.), mean (SD) - Sleep quality: (I)10.18 (8.74) and 7.89 (7.17) vs. (C) 7.56 (6.82) and 6.20 (7.11), p = 0.890 - Fatigue: (I) 3.48 (2.34) and 1.85 (1.61) vs. (C) 2.50 (2.71) and 2.10 (2.86), p = 0.750 - Depression: (I) 8.79 (4.23) and 4.78 (3.56) vs. (C) 7.08 (5.38) and 6.91 (5.86), p < 0.01 - Perceived stress: (I) 6.00 (2.48) and 5.22 (2.17) vs. (C) 5.08 (3.06) and 4.45 (3.39), p = 0.770 - Adherence was 61% for the yoga group	Not Reported
Valle 2017 (25), North Carolina (Diet and Physical Activity, eHealth tools)	End-of-I: 3 m, 94.3% completed both in-person/online assessments. Post-I follow-up: 6 m, 97.1% retention for in-person and 94.3% for online measurements. Data analysis: Intention To Treat	- % Weight change, median (interquartile range (IQR)): (I-1) -0.94 (-4.42-0.12) vs. (I-2) -0.22 (-4.18-1.28) vs. (C) 0.18 (-0.71-1.73), p (I-1 vs C) = ns, p (I-2 vs C) =0.357 - Weight, kg, median (IQR): (I-1) -1.0 (-4.0-0.1) vs. (I-2) -0.2 (-3.4-1.1) vs. (C) 0.2 (-0.7-1.3), p (I-1 vs C) =0.058, p (I-2 vs. C) = 0.751	Not Reported
*La Vida Activa/An Active Life* Greenlee 2013, (26), New York (Diet and Physical Activity)	End-of-I: 6 mo., I 95%; C 85% retention Post-I follow-up: 90.5% retention by 12 mo. - Data analysis: Not Reported	- Weight change (kg), mean (SD) - I: 2.87 (3.15); C (waitlist): -1.42 (2.5) p=0.03	- Weight change (kg), mean (SD) - I: 1.76 (3.21); - C (waitlist): -2.14 (3.77)
ALIVE, Paxton 2017 (27)^c^, Texas (Diet and Physical Activity, eHealth tools)	End-of-I: 3 mo., 62% retention Post-I follow-up: None Data analysis: Intention To Treat and sub analysis among intervention completers only	Physical Activity change (mean score (SE)): - Minutes of moderate to vigorous physical activity/wk: +97 (42), p<0.01 - Sugar in g/day: +63.9 (2.7), p=ns - Fiber in g/day: +1.1 (1.1), p=ns - fruit & vegetables in cup/day: +0.3 (0.2), p=ns - Saturated fat in g/day: -0.6 (0.8), p=ns - Trans fat in g/day: -0.0 (0.1), p=ns - Carbohydrates in g/day: +8.3 (6.9), p=ns Diet track (mean score (SE)): - Minutes of moderate to vigorous Physical Activity/wk: +49 (40), p<0.01 - Sugar in g/day: -1.5 (2.5), p=ns - Fiber in g/day: +2.9 (1.1), p=ns - Fruit & Vegetables in cup/day: +0.7 (0.2), p<0.05 - Saturated fat in g/day: -1.8 (0.8), p=ns - Trans fat in g/day: -0.2 (0.1), p=ns - Carbohydrates in g/day: +11.4 (6.6), p=ns Effect size between tracks - Minutes of moderate to vigorous Physical Activity/wk: *δ* = 0.20, p<0.001 - Sugar in g/day: *δ* = 0.35, p=0.42 - Fiber in g/day: *δ* = 0.27, p=0.35 - Fruit & vegetables in cup/day: *δ* = 0.34, p=0.29 - Saturated fat in g/day: *δ* = 0.25, p=0.40 - Trans fat in g/day: *δ* = 0.30, p=0.90 - Carbohydrates in g/day: *δ* = 0.08, p=0.61	Not Reported
Stepping STONE Sheppard 2016 (28)^c^, Washington, DC (Diet and Physical Activity)	End-of-I: 3 mo., I 67%; C 75% retention Post-I follow-up: None Data analysis: Excluded non-completers	- Body weight change (mean lbs.): - I: -1.7; C: 0.4, P>0.05	None
MyHealth Smartphone Intervention Buscemi 2020 (29), Chicago (Diet and Physical Activity, eHealth tools) *Other publications* (84)	End-of-I: 6 wks, My Guide (MG) 95%; My Health (MH) 97% retention Post-I follow-up: 8 wks, MG 95%; MH 92% retention Data analysis: Completed cases	Estimated marginal means (SD) at post-intervention Daily fat sources: MG= 2.42(0.22) vs. MH= 2.38 (0.21), p=ns Interaction between MG and MH: Cohen’s *d* = 0.30, *p* = 0.030) Daily serving of fruit & vegetables: MG= 3.41(0.28) vs. MH= 3.53 (0.28), p=0.607 Weekly physical activity in MET-mins (mean (95% CI)) -Walking: MG= 301(218, 567) vs MH= 281 (149, 529), p = ns -Moderate physical activity: MG= 12 (7,35), MH= 7 (2,21), p = ns -Vigorous physical activity: MG= 2 (1,7) vs. MH= 4 (1,12), p = ns	Estimated marginal means (SD) at follow up Daily fat sources: MG= 2.36 (0.22) vs. MH= 2.20 (0.22), p= ns Interaction between MG and MH: Cohen’s *d* = 0.47, *p* = 0.009) Daily serving of Fruit & Vegetables: MG= 3.26 (0.28), MH= 3.59 (0.28), p= ns Weekly physical activity in MET-mins (mean (95% CI)) -Walking: MG= 262 (190,494) vs. MH= 203 (108, 383), p= ns -Moderate physical activity: MG= 11 (6,32) vs. MH= 76 (1,17), p = ns -Vigorous physical activity: MG= 5 (3,15) vs. MH= 2 (0,6), p = ns
Nuestra Salud (Our Health), Crane 2020 (31), Arizona (Diet and Physical Activity, eHealth tools)	End-of-I: 12 wks, I 86 % retention Post-I follow-up: None Data analysis: comparing cohen’s d effect size	Post-intervention (Cohen’s *d*, effect size): -fruit & vegetables in cup/day: d = 0.55, p = 0. 22 -Sugar intake (g/day): d= 0.51, p = 0.25 -Vegetables intake in cup/day: d= 0.72, p = 0.11 - Mins of physical activity/week: d= 0.42, p = 0.36 - Fiber intake in g/day: d= 0.40, p = 0.36 - Global symptom distress: d= 0.17, p = 0.73 - Self-efficacy for symptom management d= 0.01, p = 0.99	None
Mobile Health, Allicock 2020 (32), Dallas, Texas (Diet and Physical Activity, eHealth tools)	End-of-I: week 4, I 100%; C 100% retention Post-I follow-up: week 8, 100%; C 100% retention Data analysis: Included post-I-follow-up	Post-Intervention, Mean change (SD) Fruit & vegetables servings/day: I = 0.67 (2.35) vs. C= 0.78 (2.48), p = ns Fast-food consumption: I = -1.5 (1.98) vs. C= -1.11 (1.45), p = ns Minutes/day of moderate to vigorous physical activity I = +0.56 (28.10) vs. C= -10.95 (9.93), p = ns Sedentary daily time (hours/day) I = -4.37 (7.14), C= - 2.57 (3.39), p = ns	Post-intervention follow up, Mean change (SD) Fruit & vegetables svg: I = 0.23 (1.88) vs. C= 0.76 (3.11), p = ns Fast-food consumption: I = -1.76 (3.11) vs. C= -0.63 (1.77), ns Minutes/day of moderate to vigorous physical activity I = - 7.28 (15.15) vs. C = - 8.47 (8.09), p = ns Sedentary daily time (hours/day) I = - 3.62 (6.24) vs. C = - 0.88 (2.65), p = ns

I, Intervention; C, Control; mo., month; wk(s), week(s); mins, minutes; yrs, years; hrs, hours, F&V, fruits and vegetables; svg, servings; kcal, kilocalories; tsp, tablespoons; δ, effect size; CI, confidence interval; SD, standard deviation; ns, not significant, MET, metabolic equivalents.

a Dieli-Conwright examined change from baseline to 4 months.

b Taylor examined change from baseline to 2 months.

c Paxton, and Sheppard examined change from baseline to 3 months.

**Table 4:** Withdrawn and ongoing randomized controlled trials targeting physical activity behavioral changes in a Black and Latina breast cancer survivors (N = 5).

Trial name, identifier, location	Sample Characteristics	Target Behavior	Primary Outcomes
Physical activity in reducing metabolic dysregulation (MetD) in Obese Latina Breast Cancer Survivors. NCT03120390 *Trial Withdrawn* Southern California	Sample: 240 Latina women Stage eligibility: newly diagnosed I-III	Physical Activity	Change in MetD
Effect of Low vs Moderate-intensity Endurance Exercise on Physical Functioning Among Breast Cancer Survivors. NCT02982564 Puerto Rico	Sample: 142 Puerto Rican women Stage eligibility: 0-III	Physical Activity	Change in cardiorespiratory fitness Change in quality of life Change in functioning Change in depression Change in body image
RCT of Strategies to Augment Physical Activity in Black and Latina Breast and Prostate Cancer Survivors (ALLSTAR). NCT05176756 California, Pennsylvania and New York	Sample: 150 Black and Latina women Stage eligibility: at least 2 years from cancer diagnosis	Physical Activity	Change in daily step count
Reducing Metabolic Dysregulation in Obese Latina Breast Cancer Survivors Using Physical Activity: The ROSA Trial (33). NCT04717050 Boston	Sample: 160 Latina women Stage eligibility: newly diagnosed I-III	Physical Activity	- Change in MetD: insulin resistance - Change in MetD: visceral adiposity - Change in MetD: metabolic syndrome
Mi Vida Saludable!/My Health Life (34) NCT02780271 Trial completed; results not yet published New York	Sample: 167 Latina Stages: 0 - III	Diet/Physical Activity	Change in daily svgs of F&V Change in energy density

I, Intervention; C, Control; F&V, fruits and vegetables; svg, servings; MetD, Metabolic Dysregulation.

Among our 22 included trials, five were efficacy trials (57–60, 63), twelve were author-defined feasibility/pilot studies (61, 62, 64, 66–69, 85–89) and five were on-going trials (90, 91) [NCT03120390, NCT02982564, NCT05176756]. Among the 17 completed trials, 2 evaluated a diet intervention, 6 intervened on physical activity, and 9 targeted both diet and physical activity, but these studies differed in outcomes, intervention components, and study duration. Most completed trials (n=14) were conducted in survivors with stage I-III breast cancer, while nine trials also included stage 0 (57–59, 62, 64, 66–69), and three included stage IV breast cancers (60, 67, 87). Three studies did not report disease stage (61, 85, 89). The sample size across the 17 completed studies ranged from 20 to 246, with six trials focusing only on Black women (61, 63–65, 68, 89), five only on Latina women (57, 58, 69, 85, 87) and six including both groups (59, 60, 62, 66, 67, 86). Included trials were geographically limited to the Northeast USA (n=8, NY, NC, DC, NJ), Texas (n=4), California (n=3), Arizona (n=2), Illinois (n=2), Puerto Rico (n=1), Massachusetts (n=1) and one ongoing at three sites (California, New York, and Pennsylvania).

### Diet interventions (n = 2)

#### Efficacy trials (n=2)

The *¡Cocinar Para Su Salud!* trial was a culturally tailored dietary intervention by Greenlee et al. in 70 Latina survivors with breast cancer randomized to either a 12-week intervention arm (n=34) or the usual care arm (n=36) (57). The intervention arm included weekly nutrition education sessions with dietitians and chefs using an adaptation of a commercially available nutrition course, “Cook for Your Life” (www.cookforyourlife.org). The study culturally tailored their intervention by including cultural values (e.g., family and community), a bilingual nurse who self-identified as Hispanic/Latino and Spanish cooking sessions with a chef. The primary goal of the intervention was to test the effectiveness of the program to help women achieve and maintain the dietary behavioral guidelines. At 3-months, the intervention arm compared to controls, had significant improvements in daily servings of all fruits and vegetables (F&V), daily total caloric intake, and total dietary fat percent of daily total energy. At 12 months, maintenance of F&V intake was observed for the intervention group compared to the control arm. However, at 12 months there was no effect of the intervention on maintenance of improvements in intake of dietary fat, weight change, BMI change, and waist circumference.

Zuniga et al. conducted a 1:1 randomized trial of 153 survivors with breast cancer (125 completed the study: 51.2% Latina, 42.4% White, and 6.4% other race/ethnicity) that examined the effect of an education and culinary-based dietary intervention vs. usual care on adherence to the Mediterranean dietary pattern (58). Cultural adaptations were not reported. The primary goal of the intervention was to improve consumption of anti-inflammatory foods, spices, and herbs *via* monthly workshops over a span of 6 months that included hands-on cooking demonstration. Analysis for study completers only demonstrated increased adherence to an anti-inflammatory dietary pattern driven by behavioral changes in 3 out of 14 items in the Mediterranean diet recommendations (reduced intake of red meat and commercial sweets or pastries and increased servings of fish). Long-term follow-up was not conducted.

### Physical activity interventions (n = 6)

#### Efficacy trials (n = 2)

Dieli-Conwright et al. conducted a 1:1 randomized trial of 100 survivors with breast cancer (26% White, 55% Latina, 4% Black, 15% Asian/Pacific Islander) to examine the effect of a 16-week physical activity intervention arm involving three weekly sessions of supervised aerobic and resistance exercise compared to a wait list control arm on metabolic syndrome, sarcopenic obesity, and inflammatory biomarkers (59). No cultural adaptations were reported. The primary endpoint was change in metabolic syndrome z-score post intervention (4 months) with a 3-month follow-up in the intervention arm only. A favorable change in metabolic syndrome z-score, sarcopenic obesity and body composition was observed for intervention arm compared to waitlist control arm by the end of the 16-week intervention. At the 3-month follow-up, the percent of participants in the intervention arm with metabolic syndrome was unchanged.

Moadel et al. (60) conducted a study examining the effects of Hatha yoga sessions compared to waitlist control arm on quality of life among Black breast cancer survivors (n=128). The primary goal was to observe changes in quality of life. The study did not report any cultural adaptations for the intervention. Moadel et al. found an unexpected decrease in social well-being for the intervention group, although the decrease was greater in the waitlist control arm (2% vs. 13%, respectively, p <0.001). No long-term results have been reported.

#### Pilot/feasibility trials (n=4)

Mama et al. conducted Project *VIVA!*, which was a four month, three-arm, randomized pilot intervention among 89 Latina survivors with breast cancer residing in Texas or Puerto Rico (87). The trial compared the effect of a culturally adapted physical activity program and a standard physical activity program to a waitlist control arm on social cognitive theory outcomes and level of physical activity and sedentary time. The culturally tailored approach included culturally relevant images, messages and examples on the topics of self-efficacy, social modeling and social support. At 16 weeks post-intervention, there were no statistical differences between the culturally tailored and the standard physical activity intervention, but there were significant improvements from baseline to follow-up in exercise self-efficacy and physical activity intensity for both intervention arms compared to waitlist control arm. No long-term results have been reported.

Lee et al. conducted an 8-week, two-arm randomized pilot study of a high intensity interval training intervention vs. waitlist control arm on patient reported outcomes (quality of life, cancer-related fatigue, and mindfulness) and physical function among 30 patients with breast cancer (73% Latina) undergoing anthracycline-based chemotherapy (86). No cultural adaptations were reported. No statistically significant differences were found for physical activity level, weight, or BMI between groups, although researchers observed improvements in quality of life and cancer-related fatigue. Adherence was 82.3% for the intervention arm. Long-term results have not been reported.

Soltero et al. conducted an 8-week, 2-arm randomized pilot study of 20 survivors with breast cancer (65% Latina, 25% White, 5% Black, 5% Mixed race/ethnicity) (62) to compare the effect of Latin dancing (intervention arm 1) to that of Qigong/Tai Chi (intervention arm 2) on overall activity (measured daily steps with a 7-day pedometer) and body mass composition. No differences were found by intervention arm for the primary outcomes. Long-term results have not been reported.

Taylor et al. (61) conducted a study examining the effect of a Pranayama yoga intervention compared to a waitlist control arm on changes in psychosocial and functional outcomes among 33 Black breast cancer survivors. Cultural adaptions were not reported. They found significantly lower depression scores on the Center for Epidemiologic Studies Short Depression Scale (CES-D-R 10) at 2 months for the intervention arm compared to the waitlist control arm. Long-term results have not been reported.

### Combined diet and physical activity interventions (n = 9)

#### Efficacy trials (n = 1)

Stolley et al. conducted the Moving Forward trial, a lifestyle intervention for Black survivors with breast cancer that aimed to achieve a 5% weight loss (63) *via* an interventionist-guided (n=125) vs. self-guided (n=121) weight loss program focused on caloric restriction over 6 months. This study did not include a usual care arm. The Moving Forward program was culturally tailored to focus on food, family, music, social roles and relationships, and spirituality and religion for Black survivors. Compared to the self-guided arm, by 6-months the interventionist-guided arm experienced significantly greater weight loss, increase vigorous physical activity, and fiber consumption, and a decrease in daily energy and added sugars. By 12 months, the changes that persisted were improvements in physical activity, daily energy intake, and fiber and sugar consumption.

#### Pilot/feasibility trials (n=8)

We identified eight pilot/feasibility trials that incorporated both diet and physical activity into the intervention. We further subdivide this section into trials that incorporated an electronic/mobile component and trials that do not.

#### Pilot trials with an electronic/mobile component (n=5)

Ferrante et al. conducted a two-arm randomized pilot intervention among 37 Black survivors with breast cancer to examine the feasibility and efficacy of a commercially available exercise and diet self-monitoring website (SparkPeople) plus a Fitbit activity tracker (intervention arm) versus a Fitbit only waitlist control arm with a goal of 5% weight loss. No cultural adaptations were used in the study. At six months post-intervention, there was no difference in weight loss in the intervention arm versus the waitlist control arm (64). Long-term results have not been reported.

Valle et al. conducted a three-arm pilot, randomized intervention among 45 Black survivors with breast cancer that allocated participants to self-regulation of diet and exercise behaviors, daily weighing plus activity tracking (intervention arm 1), self-regulation only (intervention arm 2), and waitlist control arm over three months (65). The program focused on self-regulation of diet, exercise behaviors, and daily weighting for weight gain prevention. The study did not report use of cultural adaptations. After the 3-month intervention, no differences in median weight change were observed for the intervention arms compared to the waitlist control arm. Long-term results have not been reported.

Paxton et al. conducted the *A Lifestyle Intervention Via Email* (ALIVE) trial, a two-arm randomized pilot study (dietary arm vs. physical activity arm) delivered *via* an individualized website and interactive emails among 71 survivors with breast cancer (83% Black, 11% Latina ad 6% Mixed-race) (67). Over 3 months, the diet intervention arm focused on achieving intake of ≥ 3.5 fruits and vegetable servings/day, decreasing intake of added sugars to ≤50 g/day and ≤10% of calories from saturated fat, while the physical activity intervention arm aimed on engaging participants in ≥150 min/week of moderate to vigorous physical per week. No cultural adaptations were used in this study. Participants in the physical activity intervention arm increased their moderate to vigorous activity to a greater extent than those in the dietary intervention arm. No differences were observed in change in dietary behaviors between the physical activity and dietary tracks post intervention. Long-term follow-up was not reported.

Buscemi et al. conducted a six-week, two-arm, pilot, randomized mobile application intervention among 80 Latina breast cancer survivors comparing the My Guide app (a health-related quality of life app) to My Health app (a lifestyle focused app), which was designed with culturally appropriate lifestyle promotion information (69). Culturally tailoring of the intervention involved obtaining feedback from a community partner organization, Latina breast cancer survivors, and physicians. It included English and Spanish materials, all written content available as an audio file and culturally appropriate healthy recipes. At 6 weeks post intervention or at 8 weeks follow-up, no significant differences between the two arms were found for fruit and vegetable intake, physical activity, or sedentary behavior. Long-term follow-up was not reported.

Allicock et al. conducted a 4-week, 2-arm randomized controlled pilot intervention to examine the feasibility and efficacy of the Creating Healthy Actions through Technology (CHAT) mobile application compared to usual care among 22 Black survivors with breast cancer (89). Cultural adaptations of the intervention were not reported. No differences between study arms were observed for fruit and vegetable intake, fast-food intake, moderate to vigorous physical activity or sedentary behavior at post intervention or at the 8-week follow-up. Adherence was high, with 72% of participants completing the program. Long-term follow-up was not reported.

#### Pilot trials without an electronic/mobile application-based component (n=3)

Greenlee et al. conducted the *La Vida Activa/An Active Life*, a randomized, wait-list controlled pilot study examining the effect of a commercially available Curves exercise and nutrition program on 5% weight loss among 33 Latina and 9 Black survivors (66). Linguistic adaptions, but not cultural, were incorporated with courses were offered in Spanish and English. Greater weight loss in intervention arms compared to the waitlist control arm, was found post intervention and at 12 months, but not at 6 months.

Sheppard et al. conducted The Stepping STONE (Survivors Taking on Nutrition and Exercise) trial, a randomized pilot trial of a 12-week culturally-tailored nutrition and supervised exercise program delivered in person and *via* phone among 31 Black women (68) with the goal of achieving 5% weight loss. To culturally tailor the intervention, researchers incorporated content on faith, spirituality, traditional/cultural foods, body image perceptions and risk-related information relevant to the Black survivor’s population. No differences in weight loss were found between the intervention and control arms. However, the intervention arm experienced a 3.6-fold increase in physical activity, improved cardiovascular fitness, and reduced total energy intake, total fat, and percent of energy from fat, as well as increased fiber intake (68). Long-term follow-up was not reported.

Crane et al. reported on the *Nuestra Salud*/Our Health, a two-arm randomized, telephone-based pilot trial involving 45 dyads composed of Latina cancer survivors, 83% of whom had breast cancer, and their caregivers (85) to evaluate the feasibility, acceptability, and efficacy of a 12-week culturally (e.g., bicultural health coach, social support) and linguistically (English and Spanish) appropriate program involving symptom management and a lifestyle intervention focused on meeting diet (2.5 or more cups of fruits and vegetables) and physical activity guidelines (at least 150 minutes of moderate to vigorous activity). None of the participants in the intervention arm met the intervention diet and physical activity guidelines. The trial had high reported acceptability and completion rate (86%). Long-term follow-up was not reported.

## Discussion

To our knowledge, this is the first scoping review of randomized dietary and/or physical activity interventions focused on Black and Latina breast cancer survivors. Overall, three efficacy and five pilot studies achieved statistically significant changes at least in one of their measured outcomes, but the diversity in outcomes makes results across studies difficult to compare. In addition, only 2 efficacy and 2 pilot studies captured long-term outcomes up to 12 months, but not beyond, limiting our ability to assess long-term benefits of these interventions. Overall, tailoring of the intervention to meet the unique cultural needs of breast cancer survivors of color across the trials included in this review was limited. Only four efficacy and four pilot studies mentioned the incorporation of a culturally tailored approach. This review highlights the need for lifestyle interventions that incorporate both diet and physical activity behaviors, fully powered efficacy trials and potentially more trials that incorporate electronic/mobile components (92) and informal sources of support/social networks (family, friends, caregivers) (93, 94) as these are important determinants/facilitators of lifestyle behavior change for Black and Latina survivors.

The exercise intervention by Dieli-Conwright and colleagues is the first randomized controlled trial intervention conducted in a majority Latina breast cancer survivor population to demonstrate efficacy in reducing the prevalence of metabolic syndrome by 63% and improving inflammatory biomarker profiles after three supervised, one-on-one exercise sessions per week for 16 weeks (59). The culturally tailored *Moving Forward* diet and physical activity program resulted in weight loss of 3%, although the *a priori* intervention goal was 5% (63). The *¡Cocinar para su Salud!* Trial was highly successful at achieving its *a priori* goal of helping participants meet dietary guidelines; and a secondary analysis of this study concluded that changes in taste and snack preferences for F&V may be the most important mediator for long-term increases in behavioral interventions in Latina women (95). However, this study did not intervene on physical activity which may explain why weight changes were not observed (57). Among the efficacy trials reviewed, only one of them (63) offered a comprehensive lifestyle program of both diet and physical activity—the main components of the ACS recommendations for cancer survivors. We believe lifestyle behavioral interventions that do not incorporate both diet and physical activity may not be maximizing the full potential that favorable changes to both lifestyle behaviors can have on weight loss and ultimately breast cancer outcomes.

Among the pilot/feasibility studies included in this review, eight of them incorporated a comprehensive behavioral change program (diet and physical activity) and cultural adaptations were incorporated into three of the pilot trials (68, 69, 85). None of these pilot trials achieved the behavioral *a priori* goal (64–69, 85, 89). The lack of success in achieving behavioral changes seen in these trials may be attributed, in part, to their small sample sizes and reduced power to detect small differences, having baseline samples with high levels of physical activity, and short intervention durations. In addition, during the design and development of behavioral change interventions for cancer survivors we must take into consideration the added burden of time, travel distance, and financial considerations of attending in person interventions (96). With the development and penetrance of technology into health care the use of mobile and electronic based tools for the delivery of cancer care, symptom monitoring, and health behavior interventions is increasing (97–99). Since there was broad variability on the different types of technology used, ranging from activity trackers as used by Ferrante and Valle (64, 65) in their pilot studies to the development of mobile apps as used by Buscemi and Allicock (69, 89), it is dificult to fully assess which electronic formats could be most beneficial. Studies evaluating the acceptability and preferences for technology use in behavior interventions among survivors with prostate and breast cancers highlight that while technology interventions seem acceptable, especially given the ubiquitous and even higher rates of use among Latino adults relative to other racial and ethnic groups (92), there is variability in survivor preferences of content and that these interventions may be more intuitive when participant’s health literacy and familiarity with technology is optimized (100, 101).

Greenlee et al. (57) and Crane et al. (85) demonstrated successful uptake and maintenance of healthful behaviors in culturally tailored interventions. Some examples of how these interventions were tailored include delivery of intervention by bicultural professionals, having content in Spanish and English, modifying lifestyle recommendations to meet participant’s cultural traditions, and incorporating sources of support (i.e., caregivers). Few interventions incorporated social support systems to help survivors with breast cancer adopt healthful behaviors, despite long standing evidence that social networks have important influences on health behaviors and decisions about health and health care, including engaging in cancer preventive behaviors (102). A secondary analysis of the *¡Cocinar para su Salud!* Trial by Greenlee et al. found that that participant’s network of family (spouse and children) and friends were perceived as high sources of support to share and engage in food-related and exercise activities, but most participants also perceived family members as a barrier to eating healthy foods (93). Studies that actively engage the participant’s network may be more successful in the adoption and long-term maintenance of lifestyle behavior changes after breast cancer.

It is difficult to assess if cultural adaptions to exercise interventions may confer superior benefits over a standard exercise intervention (87), as there is only one pilot trial conducted by Mama et al. which was null (103), but it has a small sample size (culturally adapted intervention, n=30, standard intervention, n=59, control, n=30). Furthermore, this study was conducted with Puerto Rican women residing in Puerto Rico and Mexican women residing in Texas, so these results may not generalize to all Latina women who reside in the continental US as well as those of other ethnic backgrounds. Large-scale efficacy trials with cultural adaptations to exercise with greater Hispanic/Latino ethnic representation from different geographic areas as well as among Black survivors are needed to fully understand the impact adapting content could have on changing physical activity.

While findings from this review suggest that lifestyle interventions may be effective in Black and Latina survivors with breast cancer, there are some important limitations within the published literature in this area of research. Like studies in non-minority populations, there is a lack of long-term data on the existing interventions among racial and ethnic minoritized groups on survival and long-term health benefits of exercise and healthy eating and maintenance of behaviors. In the trials reviewed, only four (57, 63, 64, 66) examined maintenance of healthy eating behaviors at 1-year post-intervention, with only two of them being efficacy trials (57, 63). Future trials should consider incorporating a maintenance assessment component. A better understanding of the long-term adoption of lifestyle behavior changes and the impact on breast cancer outcomes may facilitate the translation of lifestyle interventions into clinical practice. Although nine trials included Latina survivors, it is not clear whether three of the interventions (59, 60, 67) were available in Spanish for non-English speaking survivors or survivors who preferred speaking in Spanish for their treatment-related care. Interventions that are restricted to English-speaking Latina women may not be generalizable to the larger population of Latina survivors with breast cancer and continues to foment underrepresentation of a vulnerable subgroup of Latina women in the US. Trials included in this review included few advanced stage breast cancers which are known to be more prevalent in the Black (36% regional, 9% distant) and Latina (33% regional, 6% distant) breast cancer patient population compared to White (26-30% have regional, 5% distant) women (5, 104) and therefore limit generalizability of findings to wider samples of Black and Latina survivors. Among trials reviewed, only three randomized women with a history of stage IV breast cancer (60, 67, 87). Given the beneficial effects of diet and exercise interventions on treatment adherence (44), quality of life (44, 105, 106), physical functioning (105), and survival (107), researchers should consider expanding inclusion criteria of lifestyle interventions to patients with advanced disease, though the exercise component may need to be modified in some individuals. Additionally, most of the trials in this review did not report intent-to-treat analyses. By assessing the efficacy of an intervention based on who completes the study, rather than who is randomized, we eliminate the benefits provided by a randomized study design and potentially introduce selection bias.

According to the Clinicaltrials.gov registry, five trials in Latina and Black breast cancer survivors, patients, or women at risk of breast cancer are ongoing. Of these, one was withdrawn because the principal investigator changed institutions and one is completed, but findings have yet to be published. The three non-completed ongoing trials only intervene with physical activity and are split between Latina participants (NCT04717050, NCT02982564), and Black/Latina participants (NCT05176756), with the goal to examine change in metabolic dysregulations, various physical and mental health outcomes (cardiovascular fitness, quality of life, physical functioning, depression, and body image), and physical performance scores, respectively. The withdrawn trial (NCT03120390) also intervened on physical activity only and aimed to examine changes in metabolic dysregulation among Latina participants. Lastly, we enthusiastically await the findings for the *Mi Vida Saludable*/My Healthy Life trial by Hershman and colleagues (NCT02780271) that was completed on September 11, 2020 (90) which evaluated the synergistic effects of an in-person hands-on dietary and physical activity change curriculum and e-communication stratifies on behavior change in a 4-arm randomized controlled study (90).

There are a few notable limitations among the four on-going trials, including moderate sized samples ranging from 142-160 participants. Moreover, just one of these trials incorporates both diet and physical activity components to the intervention and none include weight loss as a primary outcome. We believe that incorporating both diet and exercise counseling in lifestyle interventions may be most optimal to achieve clinically meaningful weight loss (5%) and long-term maintenance of improved behaviors. Nonetheless, we look forward to the publication of the findings from all these trials as they may provide additional insights into the development of lifestyle interventions tailored for breast cancer survivors of color.

In alignment with the nature of scoping reviews (53, 54, 108) which is to “provide an overview of the existing evidence regardless of methodological quality or risk of bias” (55), our report provides a synthesis on the evidence on lifestyle interventions for Black and Latina women with breast cancer. We summarized published and on-going randomized lifestyle interventions, we described the populations that have been included, the type of intervention or programming content used, and the outcomes measured. We summarized each study’s findings and concluded by highlighting knowledge gaps and directions for researchers and interventionists in the development of new lifestyle behavior change trials for Black and Latina women with breast cancer. In accordance with expert guidance on reporting of evidence in scoping reviews, our report does not provide a critical appraisal (or a risk of bias assessment) of this body of evidence (53, 55, 108, 109).

In conclusion, this review highlights the immediate need for additional large-scale, multi-site, randomized clinical trials consisting of diet and physical activity behavioral interventions specifically designed for Black and Latina women diagnosed with breast cancer. Trials that remove English-language eligibility criteria and provide interventions in both Spanish and English, according to participant preference, are warranted. Diet and physical activity trialists should also consider interventions that begin at the time of a breast cancer diagnosis and are conducted simultaneously with treatment (110). Intervening immediately upon receipt of a breast cancer diagnosis may be beneficial to limit treatment-related weight gain and reduce side effects, promote timely treatment completion and adherence, and ultimately, improve survival (111, 112). The findings reported in this scoping review should be considered when designing lifestyle interventions in women diagnosed with breast cancer. Randomized trials in Black and Latina women are needed that evaluate efficacy outcomes, that have long-term follow-up, that are culturally tailored, that intervene from moment of diagnosis, and that incorporate electronic/mobile components and social networks/sources of support for survivors of color.

## Data availability statement

The original contributions presented in the study are included in the article/**Supplementary Material**. Further inquiries can be directed to the corresponding author.

## Author contributions

MSP and MLI contributed to the study conception and design. Material preparation, data collection and analysis were performed by MSP, MLI, and YMR-R. The first draft of the manuscript was written by MSP, and all authors commented on previous versions of the manuscript. All authors contributed to the article and approved the submitted version.
