# Supplementary material for: Diet and physical activity interventions in Black and Latina women with breast cancer: A scoping review
Source: Front Oncol. 2023 Mar 13;13:1079293. doi: 10.3389/fonc.2023.1079293 (PMC10040823; doi:10.3389/fonc.2023.1079293)
Supplement: Supplementary file 1 [file DataSheet_1.docx]

Diet and Physical Activity Interventions in Black and Latina Women with Breast Cancer: A Scoping Review

Margaret S. Pichardo^1,2*^, Tara Sanft^3,4^, Leah M Ferrucci^1,3^, Yaideliz M Romero-Ramos^5^, Brenda Cartmel^1,3^, Maura Harrigan^3^, Ana I. Velazquez^6^, Oluwadamilola M. Fayanju^7^, Eric P. Winer^3,4^, Melinda L. Irwin^1,3^

**Affiliations**

^1^Department of Chronic Disease Epidemiology, Yale School of Public Health, New Haven, CT, USA.

^2^Department of Surgery, Hospital of the University of Pennsylvania, Philadelphia, PA, USA.

^3^Yale Cancer Center, New Haven, CT, USA.

^4^Deparment of Medical Oncology, Yale School of Medicine, New Haven, CT, USA.

^5^University of Puerto Rico-Humacao, Humacao, PR, USA

^6^Helen Diller Family Comprehensive Cancer Center, University of California San Francisco, San Francisco, CA, USA.

^7^Perelman School of Medicine at the University of Pennsylvania, Philadelphia, PA, USA.

Appendix A. Search strategy for electronic databases

**Appendix A. Search strategy for electronic databases**

PUBMED

(Breast neoplasms[MeSH Terms]) OR (breast cancer* [Title/Abstract] OR breast neoplasm*[Title/Abstract] OR breast carcinom*[Title/Abstract] OR breast tumour*[Title/Abstract] OR breast tumor*[Title/Abstract] OR breast malignan* [Title/Abstract] OR breast cancer survivor* [Title/Abstract] or breast cancer patient* [Title/Abstract] or Survivorship[Title/Abstract] or survivor* [Title/Abstract])

AND

((Health behavior or Health Promotion or Exercise or Circuit-Based Exercise or Exercise Movement Techniques or Exercise Therapy or Sports or Physical Fitness or Fitness Trackers or Resistance training or Physical Education and Training or High-intensity interval training or Exercise Test or Weight Lifting or Overweight or Body Weight Changes or Weight gain or Weight loss or Weight Reduction Programs or Body Weight or Body Weight Maintenance or Diet Therapy or Feeding Behavior or Diet or Recommended Dietary Allowances or Nutrition Therapy or Healthy Diet or Life Style or Health Education[MeSH Terms])) OR (walk*[Title/Abstract] OR jog*[Title/Abstract] OR swim*[Title/Abstract] OR bicyc*[Title/Abstract] OR cycling[Title/Abstract] OR weight lift*[Title/Abstract] OR gymnastic[Title/Abstract] OR danc*[Title/Abstract] OR yoga[Title/Abstract] OR exercis* [Title/Abstract] OR exert*[Title/Abstract] OR sport*[Title/Abstract] OR physic*[Title/Abstract] OR fit*[Title/Abstract] OR strength[Title/Abstract] OR resistance[Title/Abstract] OR circuit[Title/Abstract] OR enduran*[Title/Abstract] OR aerob*[Title/Abstract] OR inactiv*[Title/Abstract] OR sedentary[Title/Abstract] OR weight-lifting[Title/Abstract] OR weight lifting[Title/Abstract] OR weight gain[Title/Abstract] OR weight gain prevention[Title/Abstract] OR weight loss[Title/Abstract] OR weight reduction[Title/Abstract] OR body weight maintenance[Title/Abstract] OR body weight changes[Title/Abstract] OR overweight[Title/Abstract] OR obese[Title/Abstract] OR Mediterranean diet [Title/Abstract] or diet [Title/Abstract] OR diets[Title/Abstract] OR dietary[Title/Abstract] OR nutri*[Title/Abstract] OR lifestyl*[Title/Abstract] OR life styl*[Title/Abstract] OR behav*[Title/Abstract] OR educ*[Title/Abstract] OR promot*[Title/Abstract])

AND

((Hispanic Americans OR African Americans OR Minority Health OR Minority Groups[MeSH Terms])) OR (Latina* [Title/Abstract] OR Latino* [Title/Abstract] OR Latinx [Title/Abstract] OR Hispanic*[Title/Abstract] OR Mexican*[Title/Abstract] OR Mexican-American* OR Latin-American* [Title/Abstract] OR Black*[Title/Abstract] OR African Ancestry[Title/Abstract] OR African-American* [Title/Abstract] OR Minorit* [Title/Abstract] OR Ethnic*[Title/Abstract])

AND

((Clinical Studies OR Clinical Trial* OR Randomized Controlled Trial*[MeSH Terms])) OR (Randomized [Title/Abstract] OR Randomization[Title/Abstract] OR Random allocation* [Title/Abstract] OR randomly allocated [Title/Abstract] OR Random [Title/Abstract] OR randomized controlled trial* [Title/Abstract] OR controlled clinical trial* [Title/Abstract] OR Random allocation [Title] OR double blind method [Title] OR single blind method [Title] OR Clinical trial, phase I [Title/Abstract] OR clinical trial, phase ii [Title/Abstract] OR clinical trial, phase iii [Title/Abstract] OR clinical trial, phase IV [Title/Abstract] OR double blind [Title/Abstract] OR single blind [Title/Abstract] OR clinical trial* [Title/Abstract] OR trial* [Title/Abstract] OR multicenter study [Title/Abstract] OR placebo* [Title/Abstract] OR usual care [Title/Abstract] OR usual care group [Title/Abstract] OR pilot [Title/Abstract] OR efficacy study [Title/Abstract] OR feasibility study [Title/Abstract] OR feasibility [Title/Abstract] OR quasi experimental [Title/Abstract] OR Program* [Title/Abstract] OR intervention* [Title/Abstract] OR community* [Title/Abstract] OR home-based [Title/Abstract] OR home based [Title/Abstract] OR telephone-based [Title/Abstract] OR team-based [Title/Abstract] OR telephone based [Title/Abstract] OR online [Title/Abstract] OR web-based [Title/Abstract] OR web based [Title/Abstract] OR counseling[ Title/Abstract])

NOT

(Mammography[MeSH Terms]) OR (Screening mammography [Title/Abstract] OR mammogram*[Title/Abstract])

| MEDLINE (OVID) |
| --- |
| 1. exp breast neoplasms/ |
| 2. exp breast/ |
| 3. breast.tw. |
| 4. exp neoplasms/ |
| 5. exp lymphedema/ |
| 6. exp radiotherapy/ |
| 7. (breast adj25 neoplasm$).tw,ot. |
| 8. (breast adj25 cancer$).tw,ot. |
| 9. (breast adj25 tumour$).tw,ot. |
| 10. (breast adj25 tumor$).tw,ot. |
| 11. (breast adj25 carcinoma$).tw,ot. |
| 12. (breast adj25 adenocarcinoma$).tw,ot. |
| 13. (breast adj25 ductal$).tw,ot. |
| 14. (breast adj25 infiltrating$).tw,ot. |
| 15. (breast adj25 lobular$).tw,ot. |
| 16. (breast adj25 medullary$).tw,ot. |
| 17. exp mammary neoplasms/ |
| 18. (mammary adj25 neoplasm$).tw,ot. |
| 19. (mammary adj25 cancer$).tw,ot. |
| 20. (mammary adj25 tumour$).tw,ot. |
| 21. (mammary adj25 tumor$).tw,ot. |
| 22. (mammary adj25 carcinoma$).tw,ot. |
| 23. (mammary adj25 adenocarcinoma$).tw,ot. |
| 24. (mammary adj25 ductal$).tw,ot. |
| 25. (mammary adj25 infiltrating$).tw,ot. |
| 26. (mammary adj25 lobular$).tw,ot. |
| 27. (mammary adj25 medullary$).tw,ot. |
| 28. or/1-27 |
| 29. exp Health Behavior/ |
| 30. exp Health Promotion/ |
| 31. exp exercise/ |
| 32. exp exercise therapy/ |
| 33. exp Sports/ |
| 34. exp Physical Fitness/ |
| 35. exp Diet Therapy/ |
| 36. exp Feeding Behavior/ |
| 37. exp Diet/ |
| 38. exp "Physical Education and Training"/ |
| 39. exp Life Style/ |
| 40. exp Health Education/ |
| 41. (lifestyl$ or life styl$).tw,ot. |
| 42. (health$ adj6 (behav$ or educ$ or promot$)).tw,ot. |
| 43. (exercis$ or physic$ activit$ or exert$ or physic$ fit$ or sport$).tw,ot. |
| 44. (walk$ or jog$ or swim$ or bicyc$ or cycling or weight lift$ or gymnastic or danc$).tw,ot. |
| 45. ((strength or resistance or circuit or enduran$ or aerob$ or physic$ or fit$) adj6 train$).tw,ot. |
| 46. (nutri$ or diet$).tw,ot. |
| 47. or/29-46 |
| 48. (Hispanic* or Latin*).mp. or exp Hispanic Americans/ or (Mexican Dominican Puerto Rican Cuban Central South adj2 American).mp. |
| 49. (Black* or African American).mp. or exp African Americans/ or exp African Continental Ancestry Group/ |
| 50. 48 or 49 |
| 51. Randomized controlled trials as Topic/ |
| 52. Randomized controlled trial/ |
| 53. Random allocation/ |
| 54. Double Blind Method/ |
| 55. Single Blind Method/ |
| 56. clinical trial/ |
| 57. clinical trial, phase i.pt. |
| 58. clinical trial, phase ii.pt. |
| 59. clinical trial, phase iii.pt. |
| 60. clinical trial, phase iv.pt. |
| 61. controlled clinical trial.pt. |
| 62. randomized controlled trial.pt. |
| 63. multicenter study.pt. |
| 64. clinical trial.pt. |
| 65. exp Clinical Trials as topic/ |
| 66. (clinical adj trial$).tw. |
| 67. ((singl$ or doubl$ or treb$ or tripl$) adj (blind$3 or mask$3)).tw. |
| 68. PLACEBOS/ |
| 69. placebo$.tw. |
| 70. randomly allocated.tw. |
| 71. (allocated adj2 random$).tw. |
| 72. or/51-71 |
| 73. case report.tw. |
| 74. letter/ |
| 75. historical article/ |
| 76. /73-75 |
| 77. 72 not 76 |
| 78. 28 and 47 and 50 and 77 |

| EMBASE (OVID) |
| --- |
| 1. exp breast neoplasms/ |
| 2. exp breast/ |
| 3. breast.tw. |
| 4. exp neoplasms/ |
| 5. exp lymphedema/ |
| 6. exp radiotherapy/ |
| 7. (breast adj25 neoplasm$).tw,ot. |
| 8. (breast adj25 cancer$).tw,ot. |
| 9. (breast adj25 tumour$).tw,ot. |
| 10. (breast adj25 tumor$).tw,ot. |
| 11. (breast adj25 carcinoma$).tw,ot. |
| 12. (breast adj25 adenocarcinoma$).tw,ot. |
| 13. (breast adj25 ductal$).tw,ot. |
| 14. (breast adj25 infiltrating$).tw,ot. |
| 15. (breast adj25 lobular$).tw,ot. |
| 16. (breast adj25 medullary$).tw,ot. |
| 17. exp mammary neoplasms/ |
| 18. (mammary adj25 neoplasm$).tw,ot. |
| 19. (mammary adj25 cancer$).tw,ot. |
| 20. (mammary adj25 tumour$).tw,ot. |
| 21. (mammary adj25 tumor$).tw,ot. |
| 22. (mammary adj25 carcinoma$).tw,ot. |
| 23. (mammary adj25 adenocarcinoma$).tw,ot. |
| 24. (mammary adj25 ductal$).tw,ot. |
| 25. (mammary adj25 infiltrating$).tw,ot. |
| 26. (mammary adj25 lobular$).tw,ot. |
| 27. (mammary adj25 medullary$).tw,ot. |
| 28. or/1-27 |
| 29. exp Health Behavior/ |
| 30. exp Health Promotion/ |
| 31. exp Exertion/ |
| 32. exp exercise/ |
| 33. exp exercise therapy/ |
| 34. exp Sports/ |
| 35. exp Physical Fitness/ |
| 36. exp Diet Therapy/ |
| 37. exp Feeding Behavior/ |
| 38. exp Diet/ |
| 39. exp "Physical Education and Training"/ |
| 40. exp Life Style/ |
| 41. exp Health Education/ |
| 42. (lifestyl$ or life styl$).tw,ot. |
| 43. (health$ adj6 (behav$ or educ$ or promot$)).tw,ot. |
| 44. (exercis$ or physic$ activit$ or exert$ or physic$ fit$ or sport$).tw,ot. |
| 45. (walk$ or jog$ or swim$ or bicyc$ or cycling or weight lift$ or gymnastic or danc$).tw,ot. |
| 46. ((strength or resistance or circuit or enduran$ or aerob$ or physic$ or fit$) adj6 train$).tw,ot. |
| 47. (nutri$ or diet$).tw,ot. |
| 48. or/29-47 |
| 49. (Hispanic* or Latin*).mp. or exp Hispanic Americans/ or (Mexican Dominican Puerto Rican Cuban Central South adj2 American).mp. |
| 50. (Black* or African American).mp. or exp African Americans/ or exp African Continental Ancestry Group/ |
| 51. 49 or 50 |
| 52. Clinical trial/ |
| 53. Randomized controlled trial/ |
| 54. Randomization/ |
| 55. Single blind procedure/ |
| 56. Double blind procedure/ |
| 57. Crossover procedure/ |
| 58. Placebo/ |
| 59. Randomi?ed controlled trial$.tw. |
| 60. Rct.tw. |
| 61. Random allocation.tw. |
| 62. Randomly allocated.tw. |
| 63. Allocated randomly.tw. |
| 64. (allocated adj2 random$).tw. |
| 65. Single blind$.tw. |
| 66. Double blind$.tw. |
| 67. ((treble or triple) adj blind$).tw. |
| 68. Placebo$.tw. |
| 69. Prospective study/ |
| 70. or/52-69 |
| 71. Case study/ |
| 72. Case report.tw. |
| 73. Abstract report/ |
| 74. letter/ |
| 75. or/71-74 |
| 76. 70 not 75 |
| 77. 28 and 48 and 51 and 76 |

CINAHL

MH (Breast neoplasm$ or Breast or Neoplasms OR Lymphedema or Radiotherapy OR Mammary neoplasms) or AB (Breast neoplasm$ or Breast or Neoplasms OR Lymphedema or Radiotherapy OR Mammary neoplasms) or TI (Breast neoplasm$ or Breast or Neoplasms OR Lymphedema or Radiotherapy OR Mammary neoplasms)

AND

MH (Health Behavior* or Health Promotion or Exertion or Exercise or or Therapeutic Exercise or Sports or Physical Fitness or Physical Education and Training OR Eating Behavior OR Diet OR Diet Therapy OR Life Style OR Life Style, Sedentary OR Life Style Changes OR Health Education or Physical Activity) or AB (Health Behavior* or Health Promotion or Exertion or Exercise or Therapeutic Exercise or Sports or Physical Fitness or Physical Education and Training OR Eating Behavior* OR Diet OR Diet Therapy OR Life Style OR Life Style, Sedentary OR Life Style Changes OR Health Education or Physical Activity) or TI (Health Behavior* or Health Promotion or Exertion or Exercise or or Therapeutic Exercise or Sports or Physical Fitness or Physical Education and Training OR Eating Behavior OR Diet OR Diet Therapy OR Life Style OR Life Style, Sedentary OR Life Style Changes OR Health Education or Physical Activity)

AND

MH (hispanic* or latino* or latina* or mexican* or central-american* or south-american* or latinx or african-american* or black-american* or black*) OR TI (hispanic* or latino* or latina* or mexican* or central-american* or south-american* or latinx or african-american* or black-american* or black*) OR AB (hispanic* or latino* or latina* or mexican* or central-american* or south-american* or latinx or african-american* or black-american* or black*)

AND

MH (Clinical Trial Registry OR Randomized Controlled Trials OR Clinical Trials or Single-Blind Studies OR Double-Blind Studies or Placebos or usual care) or TI (Clinical Trial Registry OR Randomized Controlled Trials OR Clinical Trials or Single-Blind Studies OR Double-Blind Studies or Placebos or usual care) or AB (Clinical Trial Registry OR Randomized Controlled Trials OR Clinical Trials or Single-Blind Studies OR Double-Blind Studies or Placebos or usual care)
